# Analysis on Outcome of 3537 Patients with Coronary Artery Disease: Integrative Medicine for Cardiovascular Events

**DOI:** 10.1155/2013/162501

**Published:** 2013-08-01

**Authors:** Zhu-ye Gao, Yu Qiu, Yang Jiao, Qing-hua Shang, Hao Xu, Da-zhuo Shi

**Affiliations:** ^1^Department of Cardiology, Xiyuan Hospital, China Academy of Chinese Medical Sciences, Beijing 100091, China; ^2^Chinese Journal of Integrated Traditional and Western Medicine, Beijing 100091, China; ^3^Beijing University of Chinese Medicine, Beijing 100029, China

## Abstract

*Aims*. To investigate the treatment of hospitalized patients with coronary artery disease (CAD) and the prognostic factors in Beijing, China. *Materials and Methods*. A multicenter prospective study was conducted through an integrative platform of clinical and research at 12 hospitals in Beijing, China. The clinical information of 3537 hospitalized patients with CAD was collected from September 2009 to May 2011, and the efficacy of secondary prevention during one-year followup was evaluated. In addition, a logistic regression analysis was performed to identify some factors which will have independent impact on the prognosis. *Results*. The average age of all patients was 64.88 ± 11.97. Of them, 65.42% are males. The medicines for patients were as follows: antiplatelet drugs accounting for 91.97%, statins accounting for 83.66%, **β**-receptor blockers accounting for 72.55%, ACEI/ARB accounting for 58.92%, and revascularization (including PCI and CABG) accounting for 40.29%. The overall incidence of cardiovascular events was 13.26% (469/3537). The logistic stepwise regression analysis showed that heart failure (OR, 3.707, 95% CI = 2.756–4.986), age ≥ 65 years old (OR, 2.007, 95% CI = 1.587–2.53), and myocardial infarction (OR, 1.649, 95% CI = 1.322–2.057) were the independent risk factors of others factors for cardiovascular events that occurred during followup of one-year period. Integrative medicine (IM) therapy showed the beneficial tendency for decreasing incidence of cardiovascular events, although no statistical significance was found (OR, 0.797, 95% CI = 0.613~1.036). *Conclusions*. Heart failure, age ≥ 65 years old, and myocardial infarction were associated with an increase in incidence of cardiovascular events, and treatment with IM showed a tendency for decreasing incidence of cardiovascular events.

## 1. Introduction

According to research, the risk factors like hypertension, diabetes mellitus, dyslipidemia, and smoking have a positive correlation with CAD. If the numbers of risk factors reduced, the incidence of CAD will decrease significantly. European Society of Cardiology (ESC), American Heart Association (AHA), American College of Cardiology (ACC), and Chinese Medical Association have published in succession clinical guidelines on angina pectoris, myocardial infarction, hypertension, and dyslipidemia, which have played an important role in improving secondary prevention of CAD. However, the implementation of the previously mentioned guidelines in clinical practice IM hospitals in China and the potential benefit of IM therapy in improving CAD prognosis remain unclear. The previous study showed that the treatment of IM can prevent restenosis after PCI [1] and potentially decrease the incidence of cardiovascular events [2]. In this study, we performed a prospective research for CAD patients who were hospitalized in cardiovascular departments in twelve hospitals in Beijing from September 2009 to May 2011 for analyzing the secondary prevention status of CAD. We also investigate the one-year following incidence of cardiovascular events with the purpose of the problem of secondary prevention and the potential role of IM.

## 2. Materials and Methods

### 2.1. Patients

3537 patients were recruited from 12 hospitals in Beijing, China. The research followed guidelines of the Declaration of Helsinki and Tokyo for humans and was approved by the Institutional Human Experimentation Committee, and an informed consent was obtained. The source of patients was shown in Table 1.

### 2.2. Inclusion and Diagnostic Criteria

*Inclusion Criteria.* These include the following:

1. a hospital with CAD (angina pectoris, myocardial infarction, heart failure, or arrhythmia);
2. complying with one of the following conditions:
  - old myocardial infarction;
  - acute myocardial infarction diagnosed in hospital;
  - coronary artery stenosis >50% confirmed by coronary angiography.

### 2.3. Evaluation Methods

The diagnostic and therapeutic statuses of CAD patients were evaluated based on relevant clinical guidelines including guidelines for the diagnosis and treatment of chronic unstable angina pectoris and myocardial infarction [3] published by Chinese Medical Association Cardiovascular Society, guidelines for diagnosis and treatment of unstable angina pectoris [4], guidelines for diagnosis and treatment of myocardial infarction [5] published by American Heart Association (AHA) and American College of Cardiology (ACC), clinical guidelines about heart failure [6, 7], guidelines for diagnosis and prevention of hypertension [8, 9], diagnosis and prevention of dyslipidemia, and National Cholesterol Education Program (NCEP) Expert Panel on Detection, Evaluation, and Treatment of High Blood Cholesterol in Adults (Adult Treatment Panel III) [10, 11].

### 2.4. Definition of Cardiovascular Events

This include the following:

- death due to any cause;
- acute myocardial infarction;
- revascularization is needed.

### 2.5. Materials

#### 2.5.1. Data Collection

Research process in accordance with the subject of the investigator's brochure. The clinical researchers were all trained and they passed the examination. The clinical data collected by an integrative platform of clinical and research was analyzed after clearing up. The treatment of IM means that the patient accepts the conventional treatment of modern medicine and the treatment of herbal medicine including both herbal-based injection and Chinese patent medicine, as well as decoction for at least 7 days in-hospital or 3 months out of hospital.

#### 2.5.2. Followup

The follow-up was mainly through telephone. Home visit will be implemented if we cannot contact patients with telephone. The latest clinical data will be used if the patient lost to followup.

#### 2.5.3. Observation Index

Demographic data, general clinical condition, drug use, and outcome of followup were observed.

#### 2.5.4. Statistics

Statistical analysis of the experimental data was carried out using SPSS (version 11.0). Patients' demographic and clinical characteristics were analyzed with descriptive analytic method and the chi-square test for categorical variables. Continuous variables were presented as mean with standard deviation, and categorical variables were expressed as frequencies with percentages. Logistic regression analysis using fractional polynomial modeling was conducted to determine the association between demographic and clinical information and mortality in MACEs. The criterion for statistical significance was at *P* < 0.05.

## 3. Results

### 3.1. Demographics and Clinical Characteristics

3537 consecutive hospitalized CAD patients were enrolled into the study, which was approved by the Local Ethic Committee. The average age in patients was 64.88 ± 11.97 years (range 24–96). The number of males is 2314, accounting for 65.42%. 795 patients had a history of smoking, accounting for 22.5%. The average history of smoking was 30.93 ± 12.54 years (range 1–70). 327 patients had a history of drinking. 

### 3.2. Subtype of CAD and Complicating Disease

The 3537 patients were divided into four groups through the first diagnosis which contain angina pectoris, acute myocardial infarction, arrhythmia, and heart failure. The group of angina pectoris includes 2212 patients, accounting for 62.54%. The group of acute myocardial infarction includes 836 patients, accounting for 23.92%. The group of arrhythmia includes 244 patients, accounting for 6.90%. The group of heart failure includes 235 patients, accounting for 6.64%. The status of complicating diseases was shown in Table 2.

### 3.3. Status of Treatment

Displayed in Table 3 are the numbers and rates of each medication, coronary artery revascularization and Chinese herbal medicine, which patients had received treatment of IM.

### 3.4. Prognostic Analysis

#### 3.4.1. Prognostic Status

312 patients were lost to followup, accounting for 8.82%. The average age was 70.25 ± 10.93 (range 30–90) in the 469 patients with cardiovascular events, accounting for 14.54%. The males are 289, accounting for 61.62%.

#### 3.4.2. Single-Factor Analysis

Table 4 displayed the result of single-factor analysis, in which myocardial infarction, heart failure, stroke, arrhythmia, LDL-C elevating, age ≥ 65 years, and diabetes mellitus were significantly higher in event-group: meanwhile, TG elevating and TC elevating were significantly higher in nonevent group. 

#### 3.4.3. Logistic Regression Analysis

Based on measured indexes in which *P* < 0.1 in the former analysis and IM therapy, a multiple regression equation was generated between cardiovascular events and indexes.  Table 5 displayed the result of logistic stepwise regression analysis, in which heart failure, age ≥ 65 years, and myocardial infarction were the independent negative prognostic factors for cardiovascular events, while TG elevating was the independent protective factor.

Table 6 displayed the result of logistic regression analysis, in which IM therapy showed potential tendency for decreasing the incidence of cardiovascular events.

## 4. Discussions

It is shown that the range of the age of the majority of hospitalized CAD patients' is from 60 years old to 74 years old, accounting for 41.93% in this study. Event rate of one-year followup of hospitalized CAD patients is 14.54% (469 cases), the average age is 70.25 ± 10.93 years (range 30–90). The probability of recurrent cardiovascular events in patients whose age is that greater than 65 years old is 2.4 times of the patients whose age is lower than 65 years old (18.5% versus 7.6%). It is also suggested that age is an important affecting factor of the prognosis of CAD which cannot be changed. Other studies have confirmed that age is an independent risk factor for CAD prognosis; the rate of CAD incidence and mortality is increasing with age [12–14].

Epidemiological and clinical studies have demonstrated that hypertension, diabetes, dyslipidemia, smoking, obesity, and other risk factors can aggravate atherosclerosis and increase the morality of CAD [15]. It is shown that hypertension, diabetes, dyslipidemia, cerebrovascular disease and COPD are common complications of CAD, and with the development of CAD and the increase of age, complications are more and more serious. One research from abroad shows that once artery atherosclerosis forms, only a simple monitoring of risk factors cannot fully control the progression of disease [16]. The correlative guidelines of CAD also recommend that patients with CAD should insist on long-term drug therapies that include *β*-receptor blockers, antiplatelet agents, and statin. Overall treatment modalities in this study were similar to the suggestion of relevant guidelines. Although the treatment rate of statins, *β*-receptor blockers, and ACEI/ARB is more than 50%, neither of these treatments show beneficial effects to decrease the incidence of cardiovascular events. This result is different from previous RCTs; it may be related to the type of design and cases as well as too short follow-up period.

Therefore, as for CAD patients who also have complications, they should pay more attention to the treatment of secondary prevention drugs and to the control of the complications on the long term, for reaching the maximum limit to decrease the incidence of cardiovascular events [17]. 

Diabetes mellitus was a CAD risk equivalent [18, 19]. It is shown that diabetes and injection of insulin may be attributed to more incidences of cardiovascular events. It may contribute to serious complex illness and poor glycemic control usually accompanying CAD patients who also contract diabetes. It is certificated that the rate of cardiovascular events such as morality in CAD complicating diabetes patients is greater than that in nondiabetes ones in interrelating reach. 

It was found that patients in normal left ventricular ejection fraction group and lower left ventricular ejection fraction group have similar prognosis in previous epidemiological investigations and observational studies [20]. But most of those experiment objects have dilated cardiomyopathy, and hypertensive heart disease patients take low proportion in patients with CAD. Moreover most of the clinical trials will usually exclude patients with heart failure whose left ventricular ejection fraction is normal. CAD is one of the important causes of heart failure, and persistent coronary ischemia will further aggravate heart failure. This study also showed that heart failure can increase the incidence of endpoint events. It is important to effectively prevent HF readmissions and improve overall outcomes [21].

In recent years, the combination of medicines rises extensively worldwide, and it is used increasingly in clinical application. IM is a unique discipline in China; it also has a wide range of applications in China [22]. This study shows that, from the results of one-year followup, although IM has no statistical significance in affecting incidence of cardiovascular events, IM therapy showed the tendency of decreasing incidence of cardiovascular events. This also shows that a single drug or treatment could not prevent multiple-factor disease such as CAD. IM has advantages in improving the clinical symptoms especially for CAD patients having diabetes, high blood pressure, lung infection, and kidney disease. It was shown that it has greater advantages in improving the quality of life for patients in previous studies [23, 24]. There are many people willing to adopt the IM treatment for cardiovascular disease [25]. 

In this study, a lengthways study design was adopted, using traditional Chinese medicine clinical research integrated research platform to investigate and analogize the clinical information and cardiovascular events in patients with CAD from cardiovascular departments of 12 hospitals in Beijing. This research reflects objectively and accurately the treatment and independent prognostic factors of hospitalized CAD patients in Beijing. There are many complications of CAD: high incidence of clinical events and a gap between clinical drugs and guidelines. Therefore, we should strengthen secondary prevention degree and health education for patients with CAD. It needs to control multiple risk factors and intervene complete complex situation to reduce the incidence of complication and the rate of endpoint events.

For the limited follow-up time, this research could not make new diseases as the endpoint, such as new-onset diabetes, new-onset kidney disease, and new-onset heart failure. As IM treatments for CAD have the advantage of being multidimensional and multi-target, further analysis of research material can include factors like the patients' quality of life, degree of symptom improvement and the different situations of occurred cases. These factors could then be combinedly and systematically evaluated to define the intended population of IM treatments, and thus be used to draw up intervening programs of IM treatments for CAD.

## Figures and Tables

**Table 1:** Source of patients.

Hospital	Case (male/female)	Percentage of total
Dongzhimen Hospital affiliated to Beijing University of Chinese Medicine	178 (111/67)	5.03%
Guanganmen Hospital affiliated to China Academy of Chinese Medical Science	564 (363/201)	15.95%
Huairou Hospital of Traditional Chinese Medicine	121 (77/44)	3.42%
Beijing Hospital of Traditional Chinese Medicine	220 (142/78)	6.22%
People's Hospital affiliated to Beijing University	107 (72/35)	3.03%
Tongzhou Hospital of Traditional Chinese Medicine	160 (98/62)	4.52%
Tongren Hospital affiliated to Capital University of Medical Sciences	260 (162/98)	7.35%
Wangjing Hospital affiliated to China Academy of Chinese Medical Science	93 (58/35)	2.63%
Xiyuan Hospital affiliated to China Academy of Chinese Medical Science	462 (304/158)	13.06%
China-Japan Friendship Hospital	632 (425/207)	17.87%
Beijing Hospital of Integrated Traditional Chinese with Western Medicine	124 (80/44)	3.51%
Anzhen Hospital affiliated to Capital University of Medical Sciences	616 (422/194)	17.42%

**Table 2:** Status of complicating disease.

Complicating disease	Number	Percentage of total
Hypertension	2398	67.80%
Diabetes mellitus	1162	32.85%
Dyslipidemia	1161	32.82%
Old myocardial infarction	741	20.95%
Stroke	496	14.02%
Chronic obstructive pulmonary disease (COPD)	397	11.22%
Peripheral vasculopathy	165	4.66%
Chronic kidney disease	154	4.35%

**Table 3:** Status of treatment.

Treatment	Number	Percentage of total
IM therapy	1459	41.25%
PCI or CABG	1425	40.29%
Antiplatelet agents	3253	91.97%
Statins	2959	83.66%
Nitrate medications	2709	76.59%
*β*-receptor blockers	2566	72.55%
Heparin	1905	53.86%
ACEI and ARB	2084	58.92%
Calcium channel blockers	1137	32.15%
Insulin	471	13.32%

**Table 4:** Single-factor analysis.

Variable	Nonevent group	Event group	*P*
Myocardial infarction	39.15%	47.97%	<0.001
Heart failure	6.91%	20.47%	<0.001
Stroke	4.63%	8.53%	0.001
Arrhythmia	18.71%	24.73%	0.003
TG elevating	38.90%	30.79%	0.001
LDL-C elevating	22.27%	27.39%	0.028
TC elevating	23.97%	17.57%	0.004
Age ≥ 65 years	49.28%	68.87%	<0.001
Diabetes mellitus	16.36%	20.68%	0.024
*β*-receptor blockers	73.11%	68.87%	0.059
Insulin	12.91%	15.99%	0.068
Antiplatelet agents	92.28%	89.98%	0.1
IM therapy	40.9%	43.5%	0.291

**Table 5:** Analysis of prognostic factors using multivariate logistic stepwise regression.

Variables	*β*	*P*	OR	95% CI
Heart failure	1.310	<0.001	3.707	2.756–4.986
Age ≥ 65 years	0.697	<0.001	2.007	1.587–2.539
Myocardial infarction	0.500	<0.001	1.649	1.322–2.057
TG elevating	−0.259	0.02	0.754	0.595–0.956

**Table 6:** Analysis of prognostic factors using multivariate logistic regression.

Variables	*β*	*P*	OR	95% CI
IM therapy	−0.227	0.09	0.797	0.613–1.036
Myocardial infarction	0.386	0.001	1.471	1.172–1.847
Heart failure	1.308	<0.001	3.7	2.737–5.002
Stroke	0.392	0.095	1.48	0.935–2.342
Arrhythmia	0.084	0.536	1.088	0.833–1.42
Antiplatelet agents	−0.115	0.591	0.892	0.587–1.355
*β*-receptor blockers	−0.077	0.548	0.926	0.72–1.19
Insulin	0.333	0.056	1.396	0.991–1.966
TG elevating	−0.208	0.099	0.812	0.635–1.04
LDL-C elevating	0.036	0.846	1.036	0.722–1.487
TC elevating	−0.255	0.206	0.775	0.522–1.15
Age ≥ 65	0.574	<0.001	1.775	1.384–2.278
Different hospital	0.295	0.16	1.343	0.89–2.026
Diabetes mellitus	0.072	0.647	1.075	0.789–1.465
